# Ecological and economic benefits of planting winter rapeseed (*Brassica rapa* L.) in the wind erosion area of northern China

**DOI:** 10.1038/s41598-019-56678-3

**Published:** 2019-12-30

**Authors:** Li Ma, Xuefang Wang, Yuanyuan Pu, Junyan Wu, Jeffrey A. Coulter, Xuecai Li, Lijun Wang, Lijun Liu, Yan Fang, Zaoxia Niu, Jinli Yue, Jing Bai, Yuhong Zhao, Jiaojiao Jin, Yu Chang, Wancang Sun

**Affiliations:** 1 Gansu Provincial Key Laboratory of Aridland Crop Science, Gansu Agricultural University, Lanzhou, 730070 China; 2 College of Agronomy, Gansu Agricultural University, Lanzhou, 730070 China; 3 Agriculture Technology Department, Gansu Agricultural Technology College, Lanzhou, 730020 China; 4 Department of Agronomy and Plant Genetics, University of Minnesota, St. Paul, Minnesota, MN 55108 USA; 5 Agricultural Technology Extension Center of Huining County, Baiyin, 730700 China

**Keywords:** Plant breeding, Agroecology

## Abstract

Winter and early spring wind soil erosion have considerable impacts on ecosystems, human well-being and agricultural production in the low precipitation zones of northern China. Little is known about the impact of growing winter rapeseed on ecological cropping systems and the associated economic benefits in the wind erosion area. To explore the winter rapeseed cover effect, we conducted a field experiment in which we covered the soil with winter rapeseed, winter wheat and wheat stubble at different plant density levels and used the spring bare ground as the control (CK). The effects of wind erosion, the “winter rapeseed + ” multiple cropping system, and the economic benefits were compared. There was a large difference in the dry matter, the maximum water absorption, the maximum water storage, the soil evaporation and total wind erosion, the amount of sediment transported in the stratum and the wind erosion modulus. Among them, the mean wind erosion modulus of spring sowing bare land was as high as 490.9 kg·hm^−2^·h^−1^, which was 7 and 13 times that of winter wheat and winter rapeseed, respectively. As the wind speed increased from 14 to 22 m·s^−1^, from a small density to a large density, the mean wind erosion modulus decreased from 68 to 17 kg·hm^−2^·h^−1^ for winter rapeseed, and 150 to 31 kg·hm^−2^·h^−1^ for winter wheat. Total wind-erosion of sediment transport of CK was 18.6 g·m^−2^ min^−1^, which was 16 and 31 times the mean value of winter wheat and winter rapeseed, respectively. “Winter rapeseed + ” replanting peanuts, potatoes, rice, seed melons and other crops generally increased the production value by 5–74% compared with wheat and corn intercropping, which was 98–255% higher than the traditional wheat single crop. Our results suggested that the suitable area for planting winter rapeseed in northern China was approximately 3.3 × 10^6^ hm^2^, and in terms of the best economic and ecological effects, the appropriate density was 5 × 10^5^ plants·hm^−2^ in northern China. Our results indicated that Chinese winter rapeseed was the best choice for preventing wind erosion and improving ecological and economic benefits in winter and spring in northern China; additionally, winter rapeseed has important impacts on agricultural sustainability in semi-arid and arid climates.

## Introduction

The FAO-led Global Soil Partnership reports that 75 billion tonnes of soil or 10 million hm^2^ of cropland are eroded from arable lands worldwide each year; this amount of land equates to an estimated financial loss of US$400 billion per year. China has a large population and less arable land, and the major crops still need to be grown^1,2^. China is suffering from the most serious soil erosion in the world, with more than 30.7% of land classified as seriously degraded^3^. Arable land disappears at a rate of nearly 70,000 hm^2^ per year, and nearly one centimetre of topsoil is lost annually in the Loess Plateau of northern China^4,5^. In most areas of the Loess Plateau, crops are harvested once each year, and after harvest, the soil is barren for six to seven months^6^. During the non-growing season, snow covers the soil only in parts of north-western and north-eastern China. Additionally, due to the scarcity of suitable crop species or varieties that survive over winter, there is very limited cropland area covered by winter wheat (*Triticum aestivum* L.) during the winter season in north-eastern China^7^. Wind soil erosion is a major factor that contributes to soil erosion under this kind of cropping system^8^.

Wind-caused soil erosion is one of the major limiting factors affecting sustainable agriculture systems and ecological security in arid and semi-arid areas of northern China^9^. Studies have shown that has desertification affected 2.6 × 10^8^ hm^2^, or 27.5% of China’s total territory. In 2004, the amount of land area affected by desertification was 1.7 × 10^8^ hm^2^, which accounted for 18.1% of the total territory. The area of soil affected by wind erosion was 1.91 × 10^6^ km^2^ in the late 1990s. In the northwest province of Xinjiang, 82% of the eroded soil area was caused by wind^10^. Wind erosion affected more than 40% (210,000 hm^2^) of farmland in the Hexi Corridor of Gansu Province^11^. In Northeast China, black topsoil erosion also affected 38% (44,700 km^2^) of the total area^12^. The impact of wind erosion on the productivity of agricultural and terrestrial ecosystems has been well documented^2^. Soil erosion increases water runoff, thereby decreasing soil water infiltration and soil water storage capacity, causing a loss in organic matter and plant nutrients. It also affects the soil biota and overall biodiversity^8,13–15^. Regarding the off-site effects, wind erosion can accelerate the melting of glaciers and rise of snow lines^16^, increase dust pollution, and increase or decrease soil CO_2_ emissions through enhanced mineralization and sediment burial^17,18^.

Vegetative covers that protect soil loss are one of the principle parameters considered when simulating wind erosion processes^19,20^. When 60% of the soil surface is covered by vegetation, it can minimize soil loss^21,22^. During the wind erosion season, sediment loss varies based on the crops present^23^. Compared to winter wheat and winter rye, alfalfa significantly decreases wind erosion^24^. There is also a positive correlation between wind erosion and wind speed. Soil-wind erosion can be reduced with a higher height of crop stubble after harvest^11,25–27^. When this method is used, however, the problems of operational, economic, and short-term land productivity constraints remain unsolved^28,29^. In northern China, there is a shortage of economically effective winter cover crops and winter cash crops, and winter wheat does not consistently overwinter in most areas of this region^30^. Even in areas where winter wheat can overwinter, it produces a small amount of vegetative mass by winter, resulting in limited soil coverage during winter and early spring; additionally, winter wheat has a maturation period similar to that of spring wheat with little or no economic benefit^31^. Winter rapeseed can produce nearly complete soil coverage and reaches physiological maturity approximately 30 d earlier than winter wheat, which could enable multiple crops to be grown in a single growing season, with substantial economic and ecological benefits compared to winter wheat and other winter crops. However, winter rapeseed production in northern China is constrained by its climate, annual extreme low temperature of −22.7 to −45.0 °C, negative accumulated air temperature of −700 to −1,612 °C in January, and mean low temperature of −11.1 °C in the coldest month; furthermore, there are least 150 to 180 days of winter^32,33^.

Since 1996, we have studied the over-wintering problems of winter rapeseed in northern China and have developed winter rapeseed varieties with extreme cold tolerance that are adapted to this area; these varieties could serve as winter cover crops to reduce soil wind erosion while providing additional ecological and economic benefits^34,35^. The objectives of this study were to (i) compare soil wind erosion under different densities of winter rapeseed and winter wheat, as well as wheat stubble with spring sowing, in the Loess Plateau of northern China; (ii) assess the economic performance of winter rapeseed; and (iii) develop a basis and reference for the adjustment of crop structure to reduce wind erosion through winter rapeseed production.

## Results

### Ecological benefits of different cover treatments

**Figure 1:**
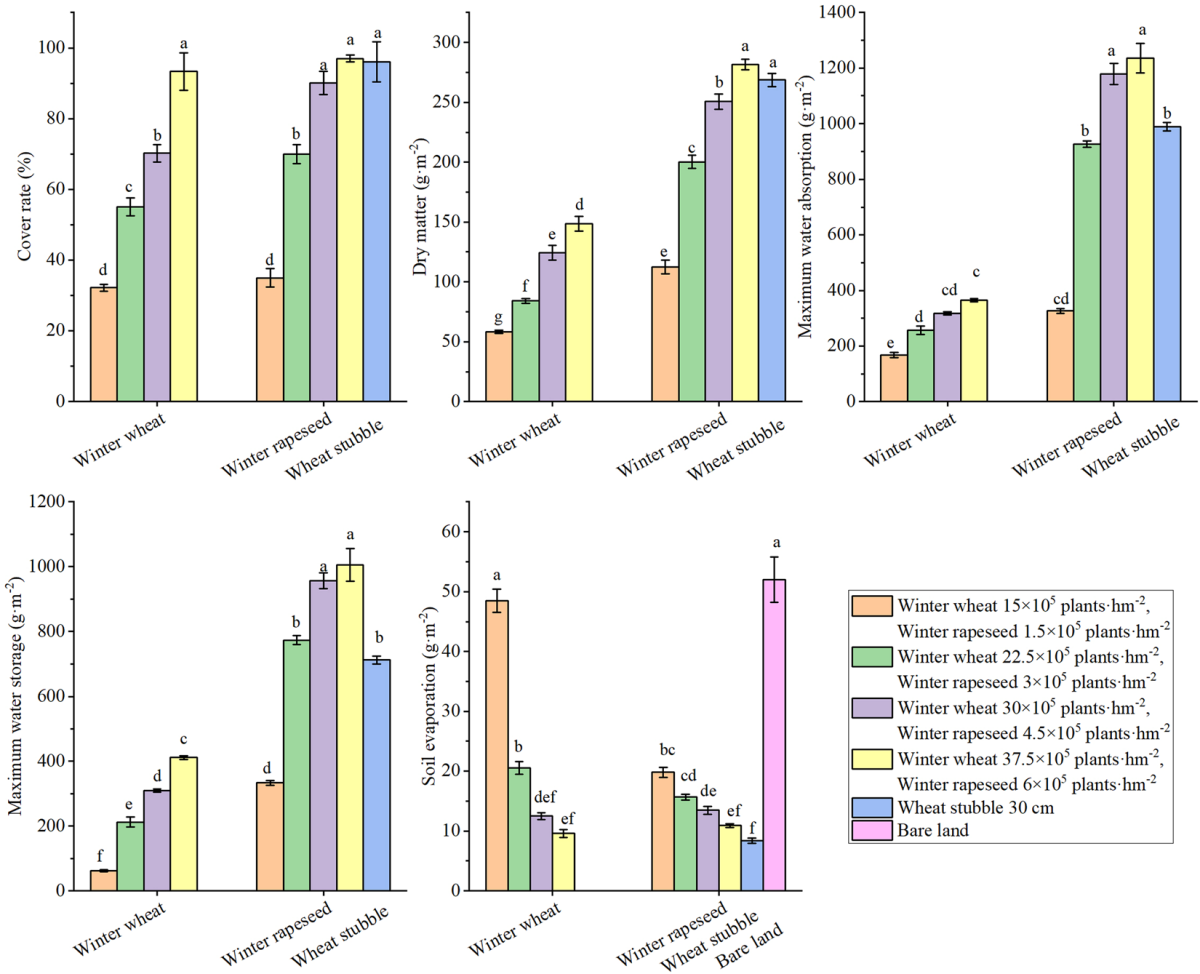
Coverage effect of different density covers. Bars with different lower case letters indicate significant differences at *P* < 0.05; error bars denote standard error of the mean (n = 3).

**Figure 2:**
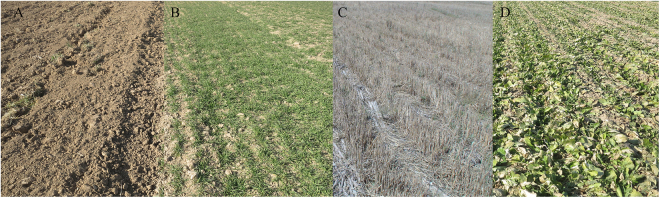
Coverage images of different treatments in the field. (**A**) bare land; (**B**) winter wheat; (**C**) wheat stubble; (**D**) winter rapeseed. Picture was taken at Wuwei city, December 2009.

As the density of the cover treatments increased, the coverage of the soil surface, cover of dry matter, maximum water absorption, and maximum water storage increased, while the soil evaporation decreased (Fig. 1). When the density of the cover treatments was increased by one level beyond the lowest density, the percentage of the soil surface covered by winter wheat and winter rapeseed increased by 22.1 and 35%, respectively; the cover of dry matter increased by 2.6 × 10^−5^ and 8.77 × 10^−5^ g·m^−2^, respectively; the maximum water absorption increased by 8.99 × 10^−5^ and 6 × 10^−4^ g·m^−2^, respectively; the maximum water storage increased by 1.5 × 10^−4^ and 4.4 × 10^−4^ g·m^−2^, respectively; and the soil evaporation decreased by 28 and 4.1%, respectively. When the density of the cover treatments increased by one level beyond the second density level, the coverage of the soil surface increased by 15.1 and 20.1%, respectively; the cover of dry matter increased by 4 × 10^−6^ and 5.05 × 10^−5^ g·m^−2^, respectively; the maximum water absorption increased by 6 × 10^−5^ and 2.52 × 10^−4^ g·m^−2^, respectively; the maximum water storage increased by 9.74 × 10^−5^ and 1.83 × 10^−4^ g·m^−2^, respectively; and the soil evaporation decreased by 39 and 14%, respectively. When the density of the cover treatments was increased further, the degree of change for these indicators decreased. Among the cover treatments, the ranges of coverage of the soil surface, cover of dry matter litter and soil evaporation were largest for wheat stubble, the ranges of maximum water absorption and maximum water storage were largest for winter rapeseed, and the ranges in these indicators with winter wheat were intermediate. To increase the amount of cover of dry matter, thereby increasing the soil water storage and water absorption and effectively reducing the soil evaporation, the effect of returning to wheat in the same year was poor, and the winter wheat produced the least cover (Fig. 2).

### Effects of winter rapeseed cover on soil erosion by wind

**Figure 3:**
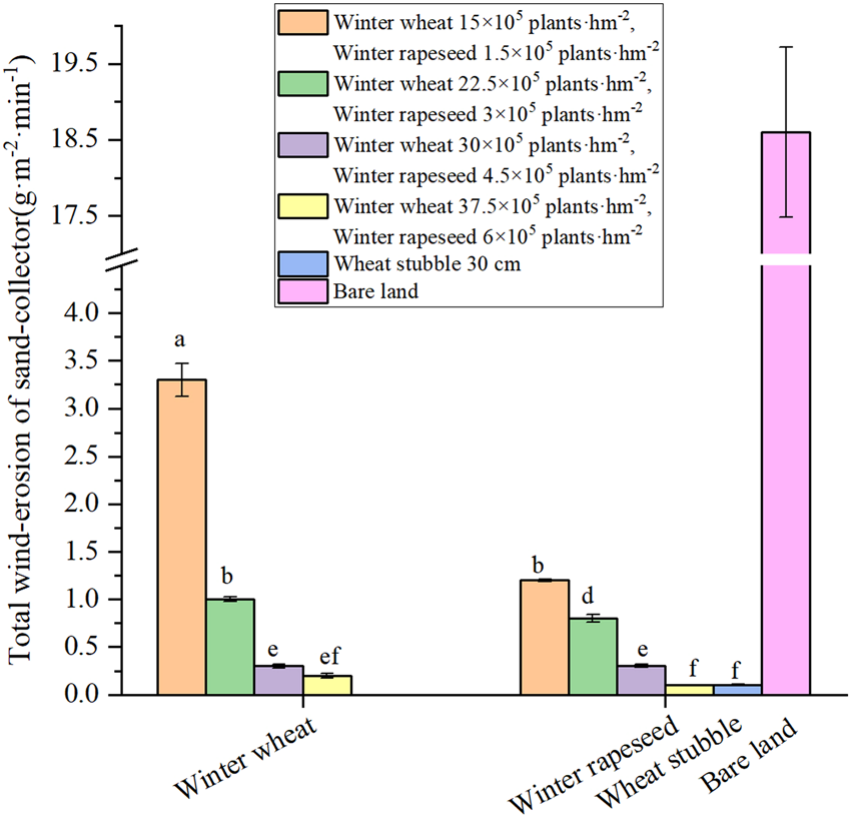
Total wind-erosion with different treatments. Bars with different lower case letters indicate significant differences at *P* < 0.05; error bars denote the standard error of the mean (n = 3).

Total wind-erosion of sediment transport in the 20-cm sand collector were affected by cover treatment and density (Fig. 3). With each increase in cover density, the total wind erosion rate was reduced by 70, 70, and 33%, respectively, for winter wheat; and by 33, 63, and 67%, respectively, for winter rapeseed. These results indicate that cover type have a relatively large effect on reducing wind erosion, while the effect of winter wheat density is large for the amount of wind erosion. Winter rapeseed can stabilize and effectively reduce soil wind erosion at lower densities. Under the higher density treatment, with the increase of density, the decrease of wind erosion of winter wheat was less than that of winter rapeseed.

**Figure 4:**
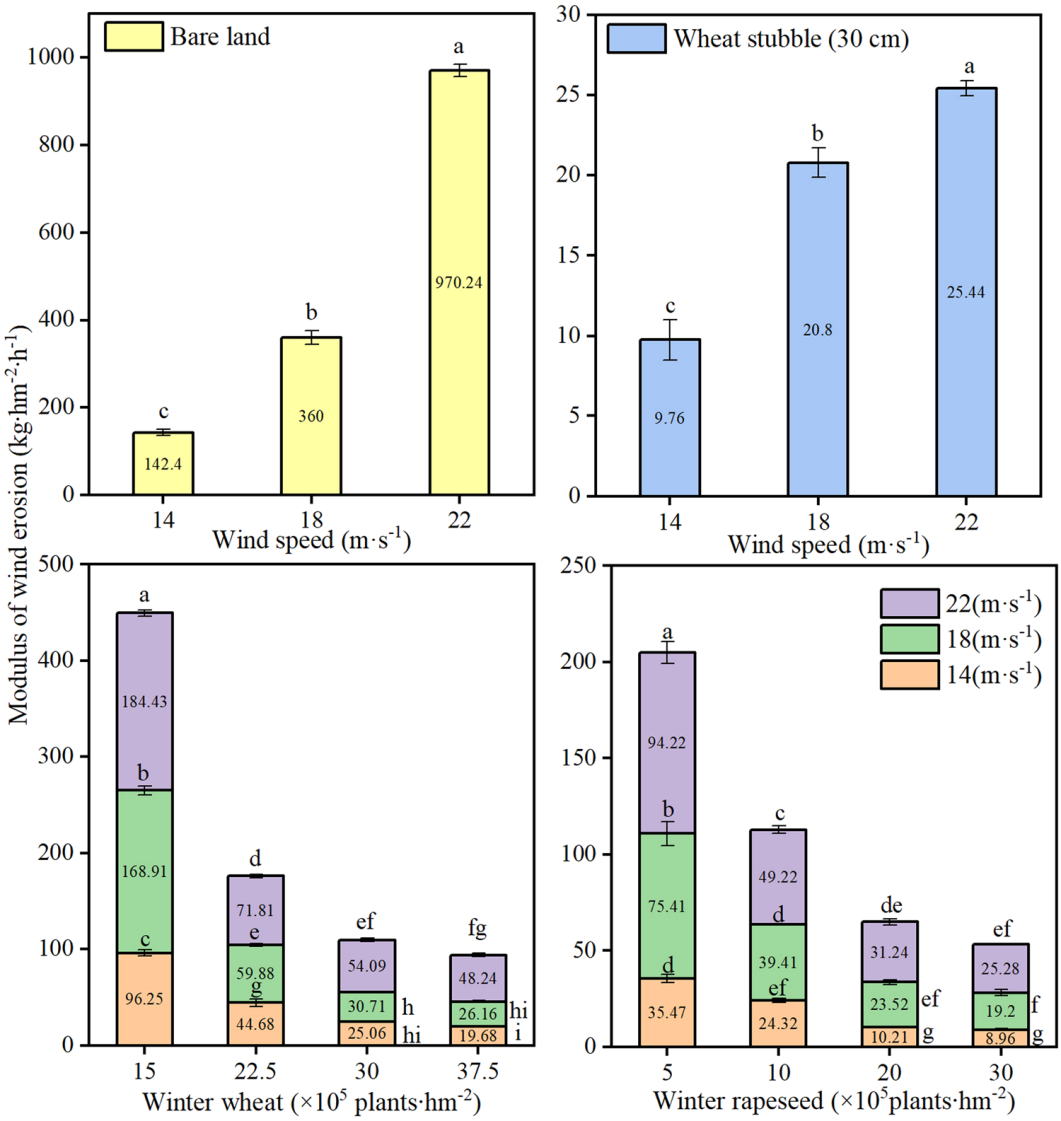
Modulus of wind erosion at different coverage densities. Numbers inside the column as the modulus of wind erosion at indicate wind speed. Bars with different lower case letters indicate significant differences at *P* < 0.05; error bars denote the standard error of the mean (n = 3).

The wind erosion modulus is an indicator of the degree of wind erosion, and the greater the wind erosion modulus is, the more serious the wind erosion is^14,36^. The degree of wind erosion at spring sowing for non-covered soil can be considerable. The mean wind erosion modulus across the tested wind speeds was 491 kg·hm^−2^·h^−1^ in the spring for non-covered soil, and this value was 10, 7, and 13 times greater than that of wheat stubble, winter wheat, and winter rapeseed, respectively (Fig. 4). The wind erosion modulus increased with wind speed, but the magnitude of the increase differed among the cover treatments. As the wind speed increased from 14 to 22 m·s^−1^, from a small density to a large density, the mean wind erosion modulus decreased from 68 to 17 kg·hm^−2^·h^−1^ for winter rapeseed and from 150 to 31 kg·hm^−2^·h^−1^ for winter wheat. This result indicates that the wind erosion resistance of wheat stubble and winter wheat was weakened with increasing wind speed, while that of winter rapeseed was enhanced with increasing wind speed. These results indicate that winter rapeseed has greater utility than winter wheat and winter stubble in terms of controlling wind erosion. At high densities, the soil surface of winter rapeseed leaves is rougher than that of wheat stubble and winter wheat as the friction and sand speed increase, thereby effectively reducing the sediment transport volume and the total amount of soil wind erosion.

### Relationship between wind erosion and soil coverage

**Figure 5:**
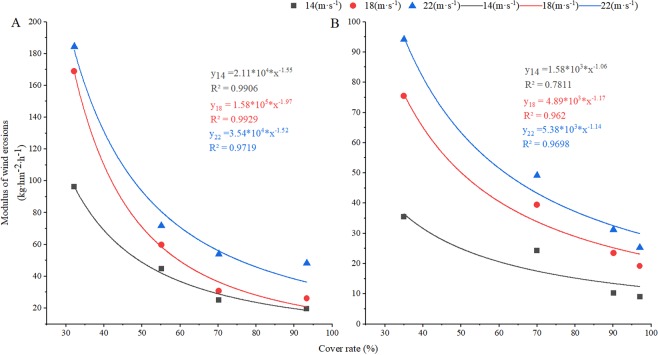
Relationship between wind erosion and soil coverage rate with different cover crops at different wind speeds. (**A**) winter wheat; (**B**) winter rapeseed.

The response between the wind erosion modulus and the coverage rate differed with wind speed and cover treatment (Fig. 5). The slope value for the relationship between the wind erosion modulus and the coverage rate of winter rapeseed was lower than that of winter wheat, indicating that the wind erosion resistance of winter rape was stronger than that of winter wheat. At a wind speed of 22 m·s^−1^, the absolute values of the slope were 1.52 and 1.14 for winter wheat and winter rapeseed, respectively. This result indicates that the underlying surface covered by winter rapeseed has a large amount of roughness and friction resistance; thus, the effect of increasing wind speed on sediment transport at different heights is relatively small, thereby reducing the wind erosion modulus more stably and effectively than winter wheat. In contrast, the change in soil coverage with winter wheat is very sensitive to the influence of the surface roughness, friction speed, sand speed and velocity, and sediment transport at different heights, resulting in the greatest changes in wind erosion modulus.

### Economic benefits of winter rapeseed coverage

**Figure 6:**
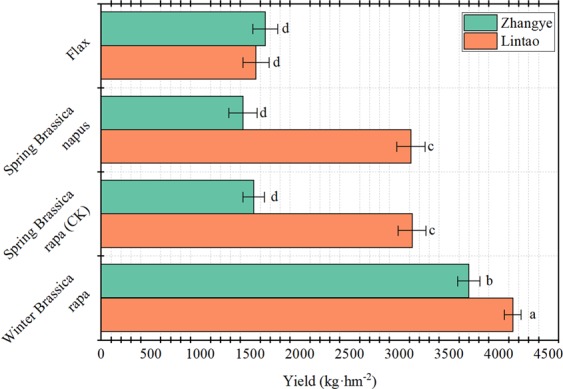
The yields of winter *Brassica rapa*, spring *Brassica rapa*, spring *Brassica napus* and flax at different locations. Zhangye city is located in the west of Gansu Province, and Lintao County is located in the central part of Gansu Province. Bars with different lower case letters indicate significant differences at *P* < 0.05; error bars denote the standard error of the mean (n = 3).

Winter *Brassica rapa* has the highest overwintering rate and yield at a density of 4.5 × 10^5^ plants·hm^−2^, ensuring that the wintering rate of winter rapeseed is the most direct way to ensure yield efficiency^32,33,35^. The yield differed significantly among winter *Brassica rapa*, spring *Brassica rapa*, spring *Brassica napus*, and flax at Lintao and Zhangye (Fig. 6). The winter *Brassica rapa* produced the greatest yield at Lintao and Zhangye (4141.7 and 3699.7 kg·hm^−2^, respectively). Compared to spring *Brassica rapa*, spring *Brassica napus*, and flax, winter *Brassica rapa* increased the yield by 68, 73, and 144%, respectively, and the maturity time was earlier, thereby allowing other crops to be planted following its harvest. In contrast, flax produced the lowest yield and had the longest growth period.

### “Winter rapeseed+” and its link with cropping systems and economic benefits

**Figure 7:**
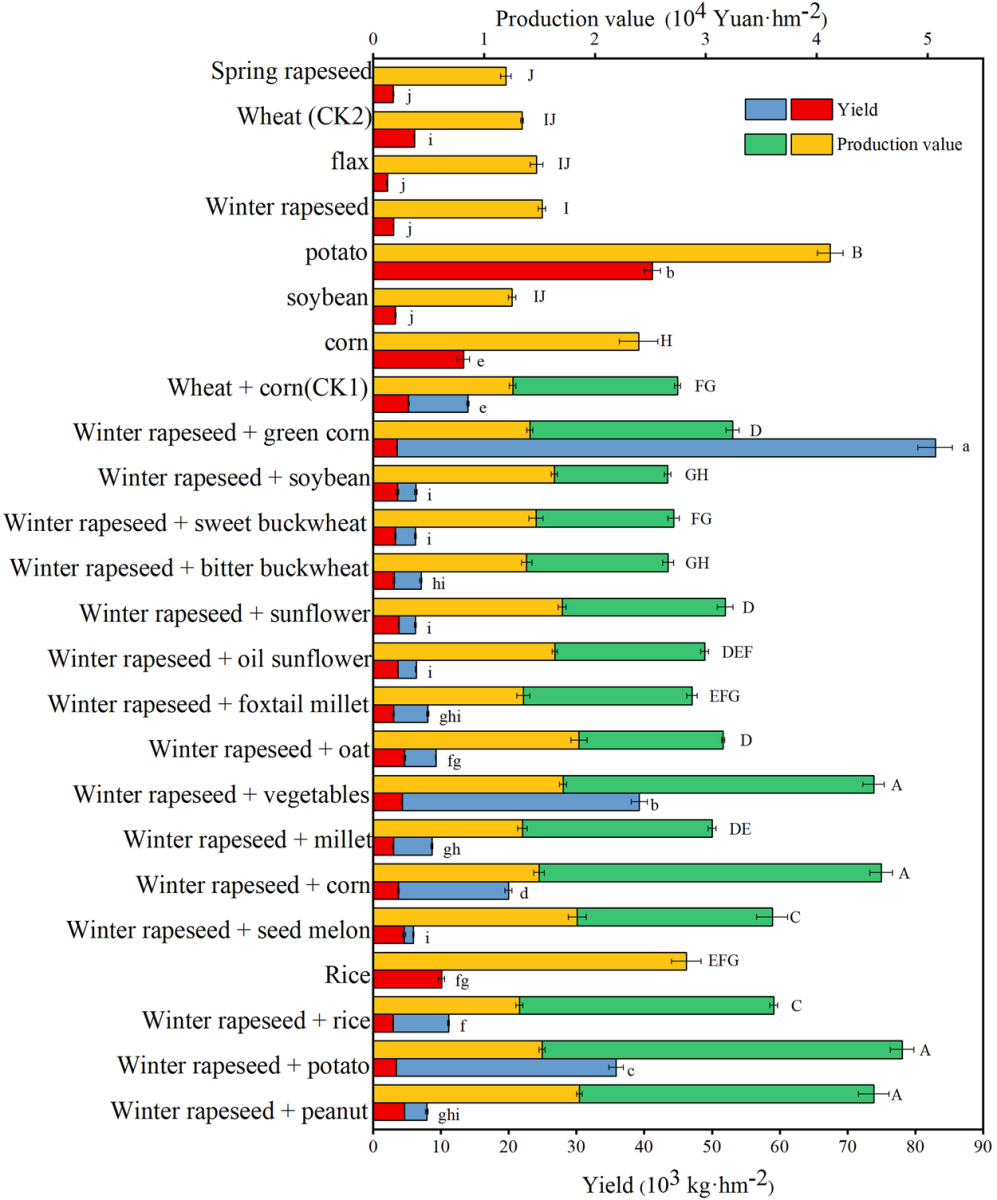
Yield and economic benefits of different cropping systems. The price of the crop is based on the local price in the second half of the year. Jingbian: rapeseed 4.5 yuan/kg, potato 1.0 yuan/kg, oil sunflower 5.0 yuan/kg; Jingyuan: rapeseed 5.0 yuan/kg, wheat 2.2 yuan/kg, flax 7.0 yuan/kg; WuZhong: rapeseed 4.0 yuan/kg, vegetables 0.8 yuan/kg, green corn 0.23 yuan/kg; Xinjiang: rapeseed 4.0 yuan/kg, corn 1.9 yuan/kg, seed melon 13.0 yuan/kg, peanuts 8.1 yuan/kg, oats 2.8 yuan/kg; Gansu: sunflower 6.0 yuan/kg; bitter buckwheat 3.2 yuan/kg, sweet buckwheat 4.2 yuan/kg, corn 1.8 yuan/kg, rapeseed 4.5 yuan/kg, foxtail millet 2.6 yuan/kg, rice 2.8 yuan/kg; Beijing: rapeseed 4.5 yuan/kg, corn 1.7 yuan/kg, soybean 3.8 yuan/kg, wheat 2.4 yuan/kg. Different lower case letters indicate significant differences of total yield at *P* < 0.05; different uppercase letters indicate significant differences of total production value at *P* < 0.05; error bars denote the standard error of the mean (n = 3).

Across several years and locations in northern China, the economic performance of “winter rapeseed+” annual double-crop and two-year three-crop crop production systems, winter rapeseed multi-cropped oil peanuts, oil sunflower, corn, rice, millet, oats, hazelnuts, seed melons, vegetables, and other crops and multiple varieties of sweet, bitter buckwheat, soybean, and green corn were evaluated (Fig. 7). There were significant differences among wheat, corn, potato, soybean, wheat, flax, winter rapeseed, and spring rapeseed. In addition, there were significant differences among winter rapeseed double-cropped peanuts, potatoes, rice, seed melons, and multi-cropped corn, hazelnuts, vegetables, oats, millet, oil sunflower, sunflower, sweet buckwheat, soybean, green corn, and monoculture. Among them, winter rapeseed multiple cropping peanuts, potatoes, rice, seed melons, and other crops generally increased the production value by 5 to 74% compared with wheat and corn intercropping, which was 98 to 255% greater than that with the traditional one-year single crop wheat, and the benefits of single winter rapeseed were slightly higher than that of wheat. Obviously, winter wheat is harvested late in northern China; thus, it is difficult to add multiple crops. Therefore, cold-resistant winter rapeseed varieties are optimal for winter and spring soil coverage and protection against wind erosion in northern China, and they enable the establishment of annual double-crop and two-year three-crop new farming systems to greatly improve the economic benefits of agriculture.

## Discussion

Farmland wind erosion hazards have been the most serious ecological environmental disaster in northern China, and farmland vegetation coverage is the determinant of farmland wind erosion^16^. Due to the strong winds and low precipitation during winter and spring, the soil texture is loose, the surface coverage lacks vegetation, and the soil moisture content is low. The combined superposition of these unfavourable factors determines that spring is the season with a high incidence of wind erosion hazards in this area. From the dynamic trend of surface coverage and the trend of wind erosion, there are changes in the characteristics of “space-time dislocation”. That is, the most severe period of wind erosion is the season with the lowest coverage in winter and spring and the season with the weakest wind erosion resistance. The direct result of this “space-time dislocation” is soil wind erosion and major economic losses^37^. The research results showed that when farmland was covered with vegetation, such as winter rapeseed, winter wheat, and wheat stubble, the soil erosion by wind was significantly reduced, thereby reducing the loss of soil organic mass, soil nutrients, and soil biota and reducing the soil water evaporation^38–43^. Our previous results showed that overwinter rapeseed had the greatest effect on soil erosion by wind among the vegetation covers. The soil surface had a higher roughness and a threshold wind velocity, as well as a lower modulus of wind erosion and a lower soil transportation rate when covered with winter rapeseed^39,43^. Soil erosion by wind is also affected by other factors, such as the density or populations of cover crop or residues (e.g., stubble, stalk, mulch). Vegetation characteristics, such as density, stalk height, and diameter per unit soil area, determine the silhouette area where the wind must pass. When the silhouette area index was increased, wind speeds above the soil surface were reduced, and soil erosion by wind was decreased^40,44^. Compared with uncovered soil, there were significant increases in soil coverage, dry mass, soil water content with wheat stubble, different densities of winter wheat and winter rapeseed covered soil, and significantly reduced soil water evaporation and wind erosion (Figs. 1–3). There were corresponding changes in the above characteristics when the stubble height and density were increased. The research results reported that when the stubble height was 31 cm and the straw mulching quantity was 3,840 kg·hm^−2^, the erosion was reduced by 88.25%, and the grain yield increased by 14.89% compared to the CK. This reduction was one of most appropriate levels of stubble height and straw mulch for crop fields in northern China^40^. Our results showed that when the stubble height was 30 cm, the erosion was reduced (indicated by the total wind erosion, modulus of wind erosion, Figs. 2 and 3) by 90%, reaching the best coverage and reducing the soil evaporation. This result was very similar to the reported appropriate levels of stubble height^40^. Similar to stubble height, winter wheat requires a highest density to have the best coverage effect, but overall, the coverage affect was much lower than that of stubble. For winter rapeseed, however, a lower density (5 × 10^5^ plants·hm^−2^) achieves a significant of a coverage effect as that of the 30 cm stubble. Our previous results showed that our best winter rapeseed varieties had an approximately 90 to 95% winter survival ratio; therefore, if planting with a density of 5 × 10^5^ plants·hm^−2^, after winter, 4.5 × 10^5^ to 4.75 × 10^5^ plants·hm^−2^ plants survived. Our previous results also showed that for the best yield, the 4.5 × 10^5^ to 5.5 × 10^5^ plants·hm^−2^ was the optimal density in different regions of northern China^32,33,35,40^ (Fig. 6), which was consistent with after the winter survival plant density. Together with these reports, our results indicated that for winter rapeseed coverage, the appropriate density was 5 × 10^5^ plants·hm^−2^ in northern China. The wind erosion modulus is an indicator of the degree of wind erosion, and the greater the wind erosion modulus is, the more serious the wind erosion is^14,36^. The effects on the prevention of soil erosion in winter rapeseed with deferent densities were very similar in wheat stubble with different heights, except for the modulus of wind erosion, which significantly decreased in winter rapeseed compared with stubble (Figs. 2 and 3). This result confirmed our previous result that showed winter rapeseed coverage had the most efficient effect on soil erosion by wind^39,43^.

Agroecosystems that are not planted during winter remain bare and unproductive for up 6 to 7 months of the year^6^. There is a shortage of economically effective winter cover crops and winter cash crops in northern China^30^. To address these problems, we have developed winter rapeseed varieties with extreme cold tolerance that are adapted to this area, and these varieties could serve as winter cover crops to reduce soil wind erosion while providing additional ecological and economic benefits^34,35^. Our results showed that the changes in the main indicators, such as the coverage ratio, soil water content and wind erosion modulus, were consistent with the results of other studies^39,43,45^. Our results show that the coverage effect of winter rapeseed is better than that of winter wheat and wheat stubble. Although the higher wheat stubble has a better effect of preventing wind erosion^39,40,43^, due to the toxicity and degradation of stubble when it is incorporated into the soil, non-economic effects, and stubble, the spring sowing and field management work that affects the leap year, these factors have limited stubble utilization. Winter wheat can effectively reduce soil wind erosion at a higher density, but the maturity is similar to that of spring wheat, and it still continues in the cropping mode, which cannot fully utilize land resources to improve economic returns. Additionally, at the appropriate density, winter rapeseed showed a higher yield than that of other oil seed crops (Fig. 5). Therefore, winter rapeseed is the best cover crop in the region, and it can be used to effectively solve the problem of farmland soil wind erosion. At the same time, the winter rapeseed litter easily decomposes, increasing the soil organic matter content and effectively utilizing the light, heat, water, soil and other resources of the area in the autumn and spring, and the maturity is early. The winter rapeseed coverage provides the temporal and spatial conditions for reforming the farming system so that the economic, social and ecological benefits are simultaneously improved.

The “Winter rapeseed+” mode is a dual-energy crop suitable for wind erosion areas in northern China. Winter rapeseed is not only an excellent winter cover crop but also an important pioneer crop for farming. Winter rapeseed matures early, as it is planted at the end of August or mid-September of the previous year, and it matures in late May of the following year. The results showed that the remaining light and heat conditions can replant corn, soybean, potato, miscellaneous grains, vegetables and other crops, thus changing the traditional one-year-old farming system to the “winter rapeseed+” one-year two-crop and two-year three-crop production systems (Fig. 7). According to research, the suitable area for winter rapeseed in northern China is approximately 3.3 × 10^6^ hm^2^^32,38,41^. Therefore, by developing the “winter rapeseed+” one-year two-crop and two-year three-crop production systems, the planting area can be increased by approximately 1.7 × 10^6^ hm^2^, achieving the synergistic growth of grain and oil. It is of great significance to solve the problem of China’s grain and oil supply, and it is suitable for wind-resistant crops in northern China.

Xinjiang, Qinghai, Tibet, Gansu, Ningxia, and northern Shanxi, north-central Shanxi, Hebei, Beijing and other northern dry and cold regions, with sufficient sunshine and vast land, are some of the areas with the most potential for agricultural production development in China^34^. However, in most areas, there is insufficient heat, the crop production is insufficient for two seasons, and there is more than one season. In autumn and winter, large areas of land are idle. Winter rapeseed is planted in early September and matures from late May to early June. Therefore, winter rapeseed production can make full use of light, heat, water and soil resources in autumn and early spring. We can reform the traditional one-year-old cropping system and establish annual double-crop and two-year three-crop production systems linked by winter rapeseed. To improve the economic index of the multiple cropping index and unit land area, we can organically combine agricultural production and ecological environment construction, which would simultaneously improve ecological benefits and economic benefits^34^. Therefore, vigorously promoting the “winter rapeseed+” model in the wind erosion area in northern China is the best choice for improving the ecological environment and economic efficiency.

## Materials and Methods

### Experimental sites

From 1996 to the present, winter rapeseed multiple cropping and its model tests were carried out in Gansu, Xinjiang, Tibet, Qinghai, Ningxia, Shanxi and Beijing in the northern wind erosion area of China. From April 2007 to October 2013, soil wind erosion studies with different coverages and different densities were carried out in Wuwei, Gansu. The total nitrogen (N), phosphorous (P), potassium (K), and organic matter at the experimental site were 0.83 g·kg^−1^, 1.27 g·kg^−1^, 238.26 mg·kg^−1^ and 13.4 g·kg^−1^ respectively.

The Agricultural Experiment Station of Gansu Agricultural University is in Huangyang town, Wuwei, Gansu Province (37°41′00′′N, 102°50′00′′E, 1760m above sea level), located at the eastern end of the Hexi Corridor, China, from April 2007 through March 2012. The annual average temperature is 7.8 °C, the extreme low air temperature is −27.8 °C, the precipitation is 200 mm, and the evaporation is 2,600 mm. The annual wind speed >6 m·s^−1^ is >200 d, and the number of days of wind over 17 m·s^−1^ (level 8) is generally from 30 to 80 d. In this region, the standard practices are to produce one crop per year and maintain a bare soil surface with no or minimal crop residues or vegetation from harvest until planting.

### Experimental design and crop management

In Huangyang towns of Wuwei city, the soil cover treatments were winter rapeseed, winter wheat, wheat stubble, and no cover (CK). After harvesting spring wheat, the land is ploughed. The winter *Brassica rapa* L. variety of Longyou-6 was planted on August 20 and had 4 treatment levels: 1.5 × 10^5^, 3 × 10^5^, 4.5 × 10^5^, and 6 × 10^5^ plants hm^−2^. The winter wheat variety of Aijiao-7 was planted on September 7 and had 4 treatment levels: 15 × 10^5^, 22.5 × 10^5^, 30 × 10^5^ and 37.5 × 10^5^ plants hm^−2^. When wheat is harvested, the height of the designed wheat stubble is kept at 30 cm for the winter. When there is no cover, planting occurs in late March of the following year. Among them, winter rapeseed and winter wheat returned to green in March of the following year. The amount of soil evaporation was determined by selecting the “S” type in the field^43^. The aboveground crops were taken in the field, and the cover rate [E = 830.14(8.20 × 10^−5^)^VCR^], where E is the wind erosion rate (g/min), VCR is the vegetation coverage or vegetation density]^46^. Maximum water absorption was the weight of the 1 m^3^ aboveground crops (winter wheat/winter rapeseed/wheat stubble) after it is soaked in water and fully absorbed], maximum water storage was the maximum water absorption minus the weight of the 1 m^3^ aboveground crops (winter wheat/winter rapeseed/wheat stubble), and the dry matter, soil evaporation of different density treatments could be determined^47,48^. Without destroying the surface structure, the soil samples of different treatments with a volume of 30 cm × 20 cm × 20 cm are measured in the soil sample box and brought back with plastic bags. With three replicates.

### Economic benefits of different planting modes

According to the main planting methods in production, the test materials are winter rapeseed: Longyou-6, Longyou-8; corn: Zhengdan-958, Fu Nong-1, Shendan-16, Shendan-16, Xinyu-10, Xinqing-1, Chaotian-1, Liaodan-39, Liaodan-2; soybean: Hefeng-50, Chundou-1, 87U-72; sunflower: 5009, 5010, Xinkui-5; rice: Japonica (Longjing-20), glutinous (Nuoyou-2); wheat: Yongliang-4; flax: Zhangya-2; potato: Kexin-2; buckwheat: Pingqiao-3, K208-02, K208-06; oats: Baiyan-8; millet: Longgu-7, Longmi-5. The materials were provided by the Gansu Provincial Rapeseed Engineering Technology Research Center. This paper has 24 seeding modes: winter rapeseed-corn intercropping, winter rapeseed-feeding corn intercropping, winter rapeseed-soybean multiple cropping, winter rapeseed-oil sunflower multiple cropping, winter rapeseed-rice sunflower intercropping, winter rapeseed-potato multiple cropping, winter rapeseed-sweet buckwheat multiple cropping, winter rapeseed-bitter buckwheat multiple cropping, winter rapeseed-millet multiple cropping, winter rapeseed-vegetable multiple cropping, winter rapeseed-millet multiple cropping, winter rapeseed-seed melon multiple cropping, winter rapeseed-peanut multiple cropping, winter rapeseed-oat multiple cropping, wheat-corn intercropping, single cropping of frontage crops for rotation: corn, soy, potato, winter rapeseed, flax, wheat, spring rapeseed, and rice. The main intercropping method is wheat-corn intercropping with CK1, and the single wheat is CK2. The area of the plot is 18 m^2^, with three replicates. After maturity, each crop in each treatment was sampled, the plot yield was calculated, and the yield and economic benefit were compared^47,48^.

### Wind erosion

A wind erosion test was conducted at the Key Laboratory of Desert and Desertification of the Chinese Academy of Sciences. Wind erosion was assessed through tunnel tests. The wind tunnel had a total length of 37.8 m, a test length of 16.2 m, a cross-sectional area of 1.0 × 0.6 m, and smooth walls constructed of multi-layer plywood with glass windows. The wind speed within the wind tunnel was continuously adjustable from 2 to 40 m·s^−1^ (turbulence intensity < 0.4%) and measured using a pitot tube and a micrometre pressure gauge^49^.

The sample box was placed 12.1 m from the inlet of the test section, and the surface of the box was flush with the bottom of the wind tunnel. The point of the horizontal distance of the rapeseed plant height was set at 20 cm. Blowing lasted for 5–10 min at net wind speeds of 14, 18 and 22 m·s^−1^. Soil particles were collected at heights of 2, 4, 6, 8, 10, 12, 14, 16, 18 and 20 cm using a WITSEG sand collector (CAS, China). Sample boxes were weighed before and after wind erosion using an electronic balance to calculate the amount of wind erosion^50–53^. The wind erosion rate (Q) was calculated as the amount of wind erosion per unit time in the test conditions. The wind erosion modulus was calculated as the amount of wind erosion per unit area per unit time at a given wind speed^11,50,52,54,55^.

### Statistical analysis

Data were analysed by SPSS 19.0 statistics software (IBM Corp., Chicago, USA). The assumptions of normality and common variance were verified based on scatterplots of residuals versus predicted values. Treatment means were compared using Duncan’s multiple range tests. Statistical significance of differences was accepted at *P* ≤ 0.05.
